# Nanostructure and Optical Property Tailoring of Zinc Tin Nitride Thin Films through Phenomenological Decoupling: A Pathway to Enhanced Control

**DOI:** 10.1021/acsanm.3c06178

**Published:** 2024-03-11

**Authors:** Caroline Hain, Krzysztof Wieczerzak, Daniele Casari, Amit Sharma, Angelos Xomalis, Patrick Sturm, Johann Michler, Aïcha Hessler-Wyser, Thomas Nelis

**Affiliations:** † Institute for Applied Laser, Photonics and Surface Technologies, BFH, Bern University of Applied Sciences, Quellgasse 21, Biel/Bienne 2502, Switzerland; ‡ Laboratory for Photovoltaics and Thin Film Electronics, EPFL, École Polytechnique Fédérale de Lausanne, Rue de la Maladière 71b, Neuchâtel 2000, Switzerland; § Laboratory for Mechanics of Materials and Nanostructures, Empa, Swiss Federal Laboratories for Materials Science and Technology, Feuerwerkerstrasse 39, Thun 3602, Switzerland; ∥ TOFWERK AG, Schorenstrasse 39, Thun 3645, Switzerland; ⊥ Nanoelectronics and Photonics Group, Department of Electronic Systems, Norwegian University of Science and Technology, Trondheim 7034, Norway

**Keywords:** zinc tin nitride, texture tailoring, microwave plasma, reactive HiPIMS, optical properties, plasma diagnostics, DFT

## Abstract

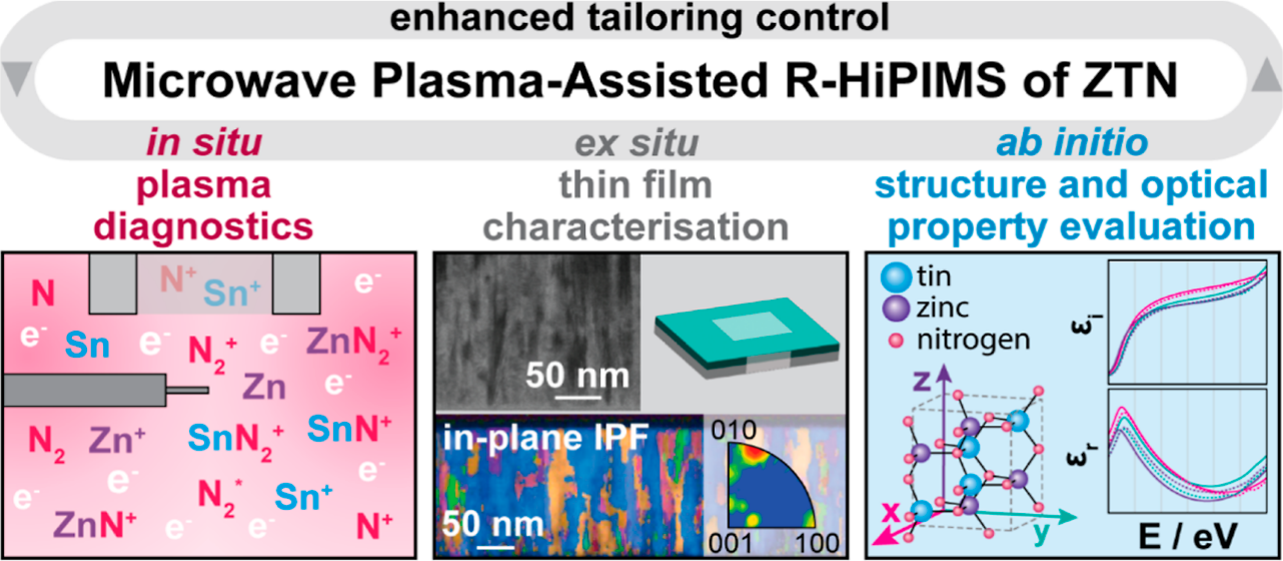

This work addresses the need for precise control of thin film sputtering processes to enable thin film material tailoring on the example of zinc tin nitride (ZTN) thin films deposited *via* microwave plasma-assisted high power reactive magnetron sputtering (MAR-HiPIMS). The applied *in situ* diagnostic techniques (Langmuir probe and energy-resolved time-of-flight mass spectrometry) supported monitoring changes in the deposition environment with respect to microwave (MW) power. During MAR-HiPIMS, the presence of nitride ions in the gas phase (ZnN^+^, ZnN_2_^+^, SnN^+^, SnN_2_^+^) was detected. This indicates that the MW plasma facilitated their production, as opposed to pure R-HiPIMS. Additionally, MW plasma caused post-ionisation of sputtered atoms and reduced the overall energy-per-charge range of incoming charged species. By varying the MW power and substrate biasing, films with comparable chemical compositions (approximately Zn_0.92_Sn_1.08_N_2_) but different structures, ranging from polycrystalline to preferentially textured, were successfully produced. The application of density functional theory (DFT) further enabled the relationship between the lattice parameters and the optical properties of ZTN to be explored, where the material’s optical anisotropy nature was determined. It was found that despite considerable differences in crystallinity, the changes induced in the lattice parameters were subangstrom, causing only minor changes in the final optical properties of ZTN.

## 1 Introduction

There is a strong need to enhance control over thin film fabrication processes to tailor the chemical composition, structure, and properties (*e.g.*, optoelectronic, mechanical) of the deposited material to meet increasingly stringent application specifications (*e.g.*, for photovoltaics, passive radiative cooling).^1−5^ The key to unlocking this tailoring potential lies in the concept of phenomenological decoupling, *i.e.*, the separation of otherwise interdependent effects. Considerable advancement has been accomplished over the years, such as the development of high-power impulse magnetron sputtering (HiPIMS),^6−9^ where, *e.g.*, average power (heat) is decoupled from the plasma density produced, or electron cyclotron resonance (ECR) microwave (MW) sources,^10,11^ where, *e.g.*, electron and ion motions are decoupled. These developments are especially relevant for the production of compound materials, where deposition is often hampered by difficulties in sourcing and sputtering compound targets (stoichiometry, purity, conductivity) or by reactive gas-metal target interactions (target poisoning, *i.e.*, the formation of a compound layer on the metallic target’s surface).^7,12−14^ In a previous work, we reported the combination of ECR MW plasma and HiPIMS for microwave plasma-assisted reactive HiPIMS (MAR-HiPIMS) of indium nitride (InN).^15^ There, it was shown that it is possible to decouple the unwanted effects of target poisoning^16^ from the reactivity of nitrogen species, the generation of nitrogen ions from the magnetron plasma, and the ion production from their energies. Pursuing this train of thought, the next step is to determine the viability of this approach during the potentially more complex process of co-sputtering different materials. The rise in complexity stems from the incomplete understanding of reactions that take place between sputtered atoms and reactive gas, posing a challenge in controlling the composition and properties of the deposited film. This investigation builds on previous research on temperature-sensitive materials,^15^ using zinc (Zn) and tin (Sn) to produce tuneable zinc tin nitride (ZTN) thin films.

In recent years, ZTN, a semiconductor belonging to the Zn-IV-N_2_ family, has attracted increasing interest in the field of optoelectronics (including photovoltaics) due to its large optical absorption coefficient and the possibility of varying the bandgap in the ultraviolet to infrared range. It is a promising candidate to replace indium gallium nitride (InGaN), as it is made from abundant and inexpensive zinc (Zn) and tin (Sn). Additionally, this material is non-toxic and recyclable and has reported bandgap values of up to approximately 2.0 eV, depending on the degree of cation disorder.^17−19^ Since the initial computational evaluation of ZTN’s (and other group-IV nitrides) optical properties as a function of the crystal structure by Paudel and Lambrecht in 2008,^20^ several groups have carried out follow-up calculations and have successfully prepared ZTN thin films by direct-current (DC) and radiofrequency (RF) reactive MS and molecular beam epitaxy (MBE) with different chemical compositions and structures.^17,19,21^ The work of Fioretti *et al.* is of particular interest as it uses a combinatorial approach to explore growth–temperature–composition relationships by producing chemically graded ZTN films at different substrate temperatures *via* RF reactive sputtering.^22^ This has allowed a wide range of structural and optoelectronic relationships to be investigated. Despite the advances in material development for optoelectronic applications, as far as the authors are aware, there are currently no studies linking the characteristics of the deposition environment to the structure of ZTN films. This is an important aspect in achieving tailoring capabilities, as it has been shown, mostly computationally, that structure can have a significant impact on the optical properties of a material.^23−26^

With the above in mind, the objective of this study was to understand how the behaviour of the deposition environment, *e.g.*, plasma potential, ion energies, and plasma chemistry (presence of Zn, Sn, and N atoms/molecules) can be influenced by the volume MW plasma and, in turn, how can this information be used to modify the nanostructure and optical properties of MAR-HiPIMS fabricated ZTN thin films of a fixed chemical composition. The power of the microwave plasma applicators and the substrate bias strategy were varied to allow for the separation of the plasma potential and ion energy during the fabrication of ZTN thin films of a fixed chemical composition. In further attempts to elucidate the effects of different synthesis conditions on the material’s optical properties as a function of film structure, calculations in the framework of the density functional theory (DFT) were performed.

## 2 Experimental Section

### 2.1 Deposition Chamber Setup

The deposition chamber was a HEXL Modular Deposition System, with two magnetrons in unbalanced configuration (Korvus Technology) installed at an angle of 27° with respect to the *z*-axis of the chamber. The chamber was evacuated by means of a HiPace 700 molecular pump (Pfeiffer Vacuum) supported by an nXDS 10i dry scroll vacuum pump (Edwards Vacuum). An in-house fabricated throttle valve positioned at the bottom of the chamber was used to vary the pumping speed. A full range compact pressure gauge (Pfeiffer Vacuum) was used to determine the pressure inside the chamber. The gas supply for Ar and N_2_ was controlled by two mass flow controllers (Teledyne Hastings Instruments, max. flows of 200 and 50 sccm, respectively). Microwave volume plasma was generated using Aura-Wave ECR coaxial plasma sources (SAIREM)^10,27^ with pulsed sputtering initiated by two HiPSTER 1 power supply units (Ionautics), in turn powered by programmable DC SL series power supplies (Magna-Power). A rotary substrate holder was attached to the lid of the chamber at a distance of 12 cm from the magnetrons. A 750 W power supply (TDK-Lambda) was connected to the holder for biasing purposes, and the grounding was fixed to the chamber’s body. Additional information about the deposition setup can be found in ref (28).

### 2.2 Deposition Environment Analysis

*In situ* diagnostics (described below) were performed during Zn and Sn sputtering in the presence of pure Ar and an Ar/N_2_ gas mixture and with and without MW plasma (0 W, 3 × 50 W, 3 × 150 W). In addition, reference measurements were made for MW plasma only.

A mixed-signal oscilloscope (Tektronix) was used to monitor the voltage and current output signals of the HiPSTER units. The data were recorded by using an averaging mode (128 pulses).

Plasma properties near the substrate were measured by using a Langmuir single probe (Impedans). The Langmuir probe consisted of a stainless-steel shaft coated with an insulated ceramic layer, with a ⌀ 0.4, 10 mm long tip installed at the end. A voltage sweep was performed from −20 to 30 V, with 0.5 V steps, and the resulting current was traced. The conditions of MAR-HiPIMS and MW plasma were measured in time-resolved and time-averaged modes, respectively. The HiPIMS pulse was set to start 20 μs after the Langmuir probe using a HiPSTER Sync Unit (Ionautics) as the external trigger.

A prototype energy-resolved time-of-flight mass spectrometer (E-ToFMS, TOFWERK AG) was used to analyse the plasma’s chemistry and ion energy distribution. This system provides mass spectra of all mass-to-charge ratios (m/Q) up to 500 Th with a mass resolving power of 500 for low m/Q ratios (Ar^2+^) and 1000 for heavier ions (^180^Hf^+^). The included electrostatic energy analyser enables determining ion energy-to-charge (E/Q) values. Ions were sampled from the gas phase into the mass spectrometer using a 20 μm orifice positioned at the height of the substrate *via* differentially pumped transfer optics. The orifice plate was grounded during measurements, and the residual pressure in the sampling ion optics was below 5 × 10^–3^ Pa. It is assumed that collisions after the sampling orifice, which could potentially lead to charge transfer or recombination reactions, were negligible.

The temperature near the substrate was measured to be approximately 100 °C during MAR-HiPIMS using a ⌀ 1 mm, 50 mm microwave plasma-resistant temperature probe (Mesurex).

### 2.3 Film Fabrication

**Figure 1:**
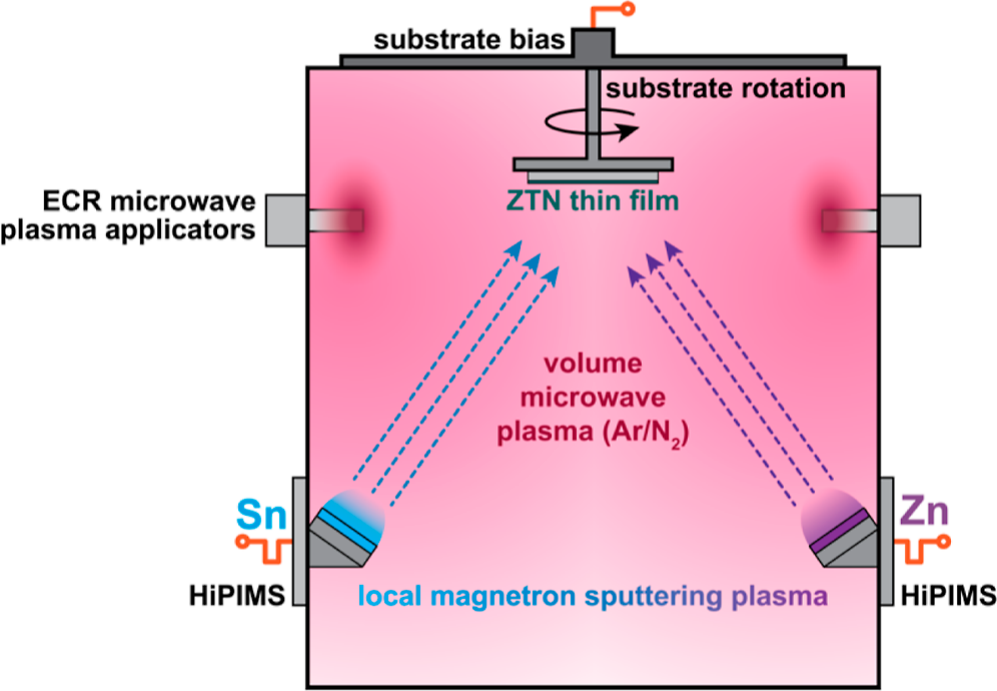
Schematic of deposition setup for MAR-HiPIMS of ZTN thin films.

ZTN thin films were fabricated using the previously described equipment by means of MAR-HiPIMS. The sputtering targets were ⌀ 50, 3 mm thick, zinc (99.995%) and tin (99.999%) (HMW Hauner GmbH & Co. KG). The substrates were ⌀ 50 mm silicon (279 ± 20 μm thick, n-type <100>-oriented from MicroChemicals) and sapphire (330 ± 25 μm thick, *c*-plane (0002) single-side polished from University Wafers) wafers. The system was evacuated until a minimum base pressure of 5 × 10^–4^ Pa was reached. To remove organic contaminants and native oxides from substrate surfaces, a sputter cleaning pretreatment step (3 × 50 W MW power, 10 sccm Ar, pressure 0.2 Pa, −150 V substrate bias) was applied for 5 min prior to film deposition. The deposition process began immediately after pretreatment without breaking the vacuum. The working gas composition (Ar/N_2_ gas flow ratio of 60/40 sccm) and pressure (0.6 Pa) remained constant throughout all of the deposition processes. The targets were pre-sputtered beneath a shutter to eliminate contaminants and/or oxides on their surface. Subsequently, the shutters were raised, and the microwave generators remained on throughout the process. The selected deposition and sputtering parameters for all produced ZTN thin film samples are summarised in Table 1. The microwave power (3 × 50 W, 3 × 150 W) and substrate bias (floating, grounded, −25 V) were varied. Uniform deposition was ensured by fully rotating the substrates at 15 rpm, while the process duration was set to 30 min to achieve film thicknesses of around 200 nm. The deposition configuration is illustrated schematically in Figure 1.

**Table 1:** Summary of Deposition and Sputtering Parameters Used during the Fabrication of all ZTN Thin Film Samples

deposition parameters used for fabricating ZTN thin films on both silicon and sapphire substrates				
pressure/Pa	gas composition	process time/min	MW power/W	substrate bias
0.6	60 sccm Ar + 40 sccm N_2_	30	3 × 50, 3 × 150	floating, grounded, –25 V

sputtering parameters used throughout all ZTN deposition processes					
target	pulse width/μs	frequency/Hz	voltage/V	peak current/A	avg. power/W
Zn	75	150	680	1	10
Sn	35	150	980	7	36

### 2.4 Material Analysis

X-ray diffraction (XRD) was carried out using a D8 Discover X-ray diffractometer (Bruker). The incident beam (Cu Kα = 1.5418 Å) was conditioned by using a Göbel mirror. Measurements were collected in a 2θ angular range from 15 to 80° in grazing incidence (GIXRD) geometry, with the incident angle fixed to 1° and a step duration of 1 s.

The films’ cross-sections and surface morphologies were imaged using a Hitachi S-4800 high-resolution cold field emission (CFE) scanning electron microscope (SEM) equipped with a secondary electron (SE) detector (Hitachi High-Tech Corporation, Japan).

The films’ chemical composition was evaluated using a Mira-3 SEM (Tescan), equipped with an Octane Plus energy-dispersive X-ray spectroscopy (EDX) silicon drift detector (EDAX). The used accelerating voltage was 5 kV, and the chemical analysis was carried out using ZAF correction.

Microstructural analysis of the thin films was performed *via* transmission electron microscopy (TEM) investigations. TEM lamellae were prepared and lifted out by using a dual-beam focused ion beam (FIB/SEM) Lyra-3 (Tescan). To minimise the amount of gallium (Ga) ion beam damage during lamellae preparation, a protective layer was initially applied on top of the film through a combination of electron-beam-induced and ion-beam-induced platinum (Pt) deposition. Trenches fabrication, lift-out, and successive polishing to 100 nm thickness were performed by operating the FIB/SEM at 30 kV and by sequentially decreasing beam currents from 4.5 nA to 150 pA. A last polishing step was performed at 5 kV and 50 pA to further reduce the thickness of the ion-induced damaged layer and Ga contamination in the lamellae.^29^ Atomic resolution images were obtained using a ThermoFischer Themis 200 G3 spherical aberration (probe)-corrected TEM operating at 200 kV. Images were taken under scanning TEM (STEM) conditions while using a high-angle annular dark field (HAADF) detector. Finally, scanning precession electron diffraction (SPED), with a step size of 3 nm and a precession angle of 0.7°, was employed to capture high spatial resolution orientation maps with a DigiSTAR system (NanoMEGAS) installed in the same aberration-corrected TEM.

For evaluating the optical properties of the deposited ZTN films, light absorption measurements were performed between 1 and 4.4 eV to fully cover their expected bandgap using a commercially available absorption spectrometer (PerkinElmer 900). While characterising ZTN films deposited on Si wafers is challenging in reflection mode (due to the semi-transparent nature of the films inducing oscillation from multilayer reflections), it was possible to measure the absorptance (logarithmic scale) in all ZTN samples deposited onto sapphire substrates in transmission mode.

### 2.5 Computational Simulations

The optical properties of selected ZTN films were calculated by the full-potential (linearised) augmented plane-wave plus local orbital (FP-LAPW + lo) method^30^ within the framework of DFT,^31,32^ as implemented in the WIEN2K code.^33^ The Perdew–Burke–Ernzerhof generalised gradient approximation (PBE-GGA, for minimisation of internal parameters and volume optimisation^34^) and modified Becke and Johnson GGA (mBJ-GGA, for electronic and optical properties^35−37^) potentials were used. The muffin-tin radii (*R*_MT_) for N, Sn, and Zn were 1.66, 2.03, and 2.03, respectively. The energy cutoff, which defines the separation of the valence and core states, was chosen as −10.0 Ry. The convergence of the basis set was controlled by the cut-off parameter *R*_MT_ × *K*_max_ = 8, where *K*_max_ is the largest reciprocal lattice vector used in the plane wave expansion within the interstitial region. The magnitude of the largest vector in the charge density Fourier expansion was *G*_max_ = 12 (a.u.)^−1^. The Brillouin zones were sampled with an 11 × 10 × 8 *k*-point mesh to minimise the internal parameters and optimise the volume, while a 23 × 21 × 19 *k*-point mesh was used for calculating the optical properties. A convergence criterion for a force of 0.5 mRy/bohr, a total energy of 0.0001 Ry, and a charge distance of 0.001 e^–^ were used for internal parameter minimisation. Lattice constants of the ZTN phase were calculated from selected X-ray diffractograms using the TOPAS 5 software,^38^ based on the Rietveld method^39^ (see Supporting Information, Table S2).

## 3 Results

### 3.1 Deposition Environment Analysis

**Figure 2:**
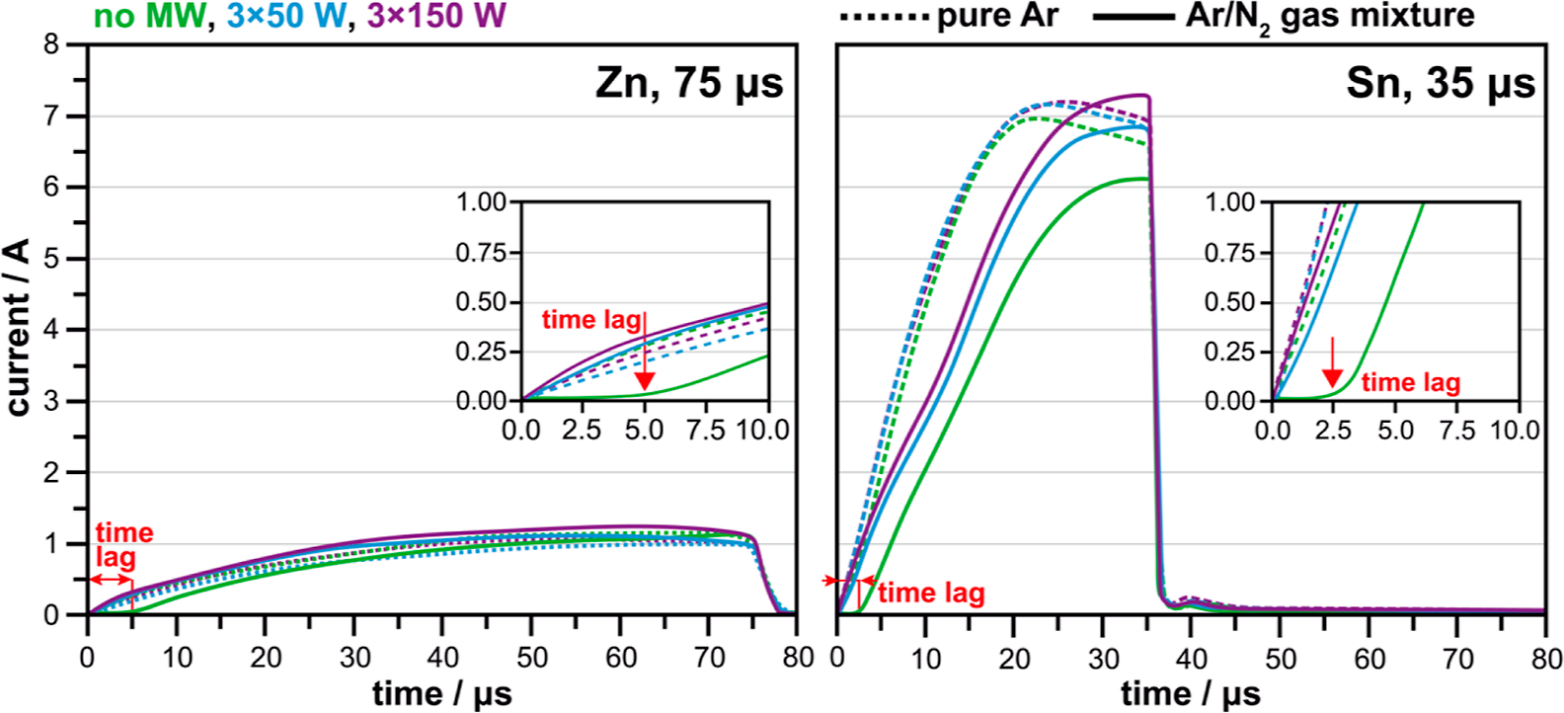
Current evolution during Zn (75 μs) and Sn (35 μs) HiPIMS using 60 sccm of pure Ar (dashed line) and a 60/40 sccm Ar/N_2_ gas mixture (full line), without MW plasma (green) and with 3 × 50 W (blue) and 3 × 150 W (purple) MW plasma, time lags marked with red arrows and highlighted in the insets.

Prior to film deposition, the nature of the zinc and tin discharges under pure Ar and mixed Ar/N_2_, without and with 3 × 50 W and 3 × 150 W MW plasma conditions was evaluated by studying the evolution in HiPIMS current waveforms (Figure 2).

Based on the obtained curves, both Zn and Sn are characterised by a similar behaviour to In.^15^ Without nitrogen, the discharge of both targets was stable with and without MW plasma. However, the addition of N_2_ caused instability in the HiPIMS discharges, resulting in a time lag of approximately 5 and 3 μs for Zn and Sn, respectively. This indicated the formation of a compound layer on the surfaces of the targets, which in turn can lead to a reduction in their SE emission yield (γ_see_).^14,16^ In the presence of MW plasma, the time lag disappears and the discharges are once again stable. Similar behaviour to MAR-HiPIMS of In was also observed in the context of Langmuir probe measurements for the plasma potential (*V*_p_), electron temperature (*kT*_e_), and plasma densities (*n*_i_, *n*_e_).^15^ The measured values for *V*_p_, *kT*_e_, *n*_i_, and *n*_e_ are listed in Table S1 of the Supporting Information.

**Figure 3:**
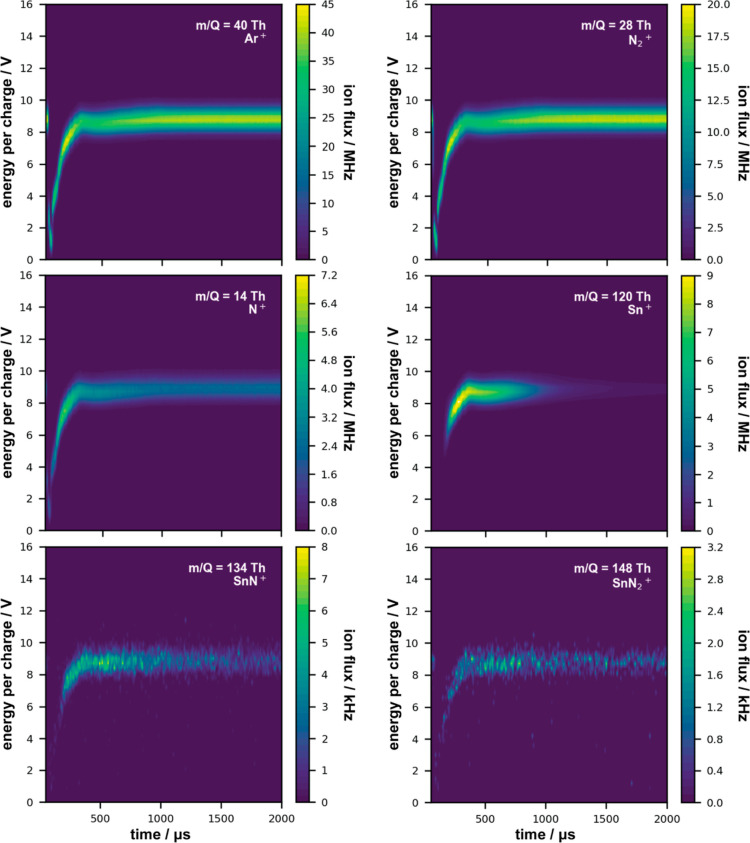
Time-resolved E-ToFMS contour plots for Ar^+^, N_2_^+^, N^+^, Sn^+^, SnN^+^, and SnN_2_^+^ ion fluxes (number of ions detected per unit of time), with their associated E/Q, obtained during MAR-HiPIMS Sn sputtering under Ar/N_2_ gas MW plasma conditions (MW power 3 × 50 W), scales adapted to data range.

**Figure 4:**
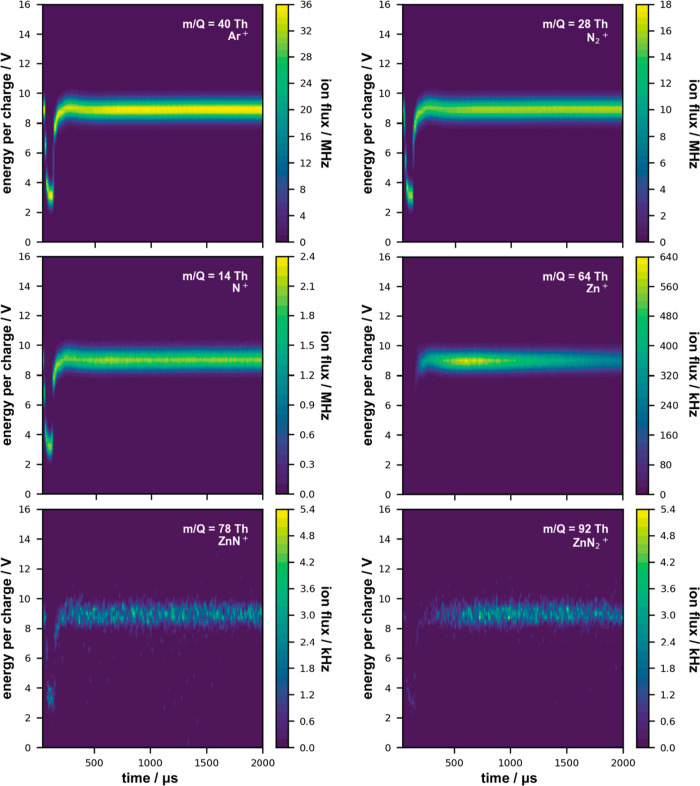
Time-resolved E-ToFMS contour plots for Ar^+^, N_2_^+^, N^+^, Zn^+^, ZnN^+^, and ZnN_2_^+^ ion fluxes (number of ions detected per unit of time), with their associated E/Q, obtained during MAR-HiPIMS Zn sputtering under Ar/N_2_ gas MW plasma conditions (MW power 3 × 50 W), scales adapted to data range.

Time-resolved E-ToFMS measurements were performed at the height of the substrate to identify the incoming ionic species, along with their E/Q. Selected results obtained during the sputtering of Sn and Zn under Ar/N_2_ gas MW plasma conditions are presented in the form of contour plots in Figures 3 and 4, respectively (the intensities are based on all isotopes of a given element). As the MW plasma is continuously on and generates charged particles throughout the chamber, the initial energy drop is caused by the high voltage applied to the magnetron, which attracts these species. After switching off the magnetron, these particles are released from the magnetron trap and can move toward the substrate region (see refs (15 and 28) for more information). By comparing the contour plots for Ar^+^, N_2_^+^, and N^+^ between the Sn and Zn discharges, both the measured E/Q values and the general temporal behaviour are similar. However, during Zn sputtering, fewer gas ions are detected, especially for N^+^ (by a factor of 3). Considering that the volume plasma conditions were the same, the cause behind the lower ionic flux is most probably related to the significantly lower peak currents employed for Zn sputtering (1 A *vs* 7 A for Sn). Therefore, the contribution of dissociated nitrogen species from the HiPIMS plasma, although not as effective as the MW plasma in the case of the material system and conditions studied, should not be ignored. The temporal behaviour of the species generated by sputtering differed depending on the pulse width used (35 μs for Sn and 75 μs for Zn). In the presence of MW plasma, ionic molecular nitrides (ZnN^+^, ZnN_2_^+^, SnN^+^, and SnN_2_^+^) were detected in the gas phase.

Comparable trends were found for the higher MW power conditions of 3 × 150 W, however, in all cases the overall E/Q distribution increased by approximately 2 V (see Supporting Information, Figures S1 and S2). For both Sn and Zn sputtering, increasing the MW power resulted in doubling of the amounts of Ar^+^ and N_2_^+^ produced. For N^+^, the effects were slightly different, where for Sn the flux increased by a factor of 1.5, whereas for Zn it tripled. No significant differences were observed for the Sn ionic flux regarding the post-ionisation of the metallic species, whereas it doubled for Zn. Similar trends were observed for the ionic molecular nitrides, except for ZnN_2_^+^, where the change in MW power did not seem to have much effect.

Additional ion flux data for R-HiPIMS without MW plasma are presented in the Supporting Information (Figures S3 and S4). Notably, no ionic nitrides were detected in the gas phase under these conditions. Moreover, the detected E/Q range for Sn^+^ and Zn^+^ during R-HiPIMS extends from 0.1 to about 5 V. The addition of MW plasma increases their E/Q but also narrows the range to 8–10 V for 3 × 50 W and 10–12 V for 3 × 150 W.

### 3.2 ZTN Thin Film Characterisation and DFT Calculations

**Figure 5:**
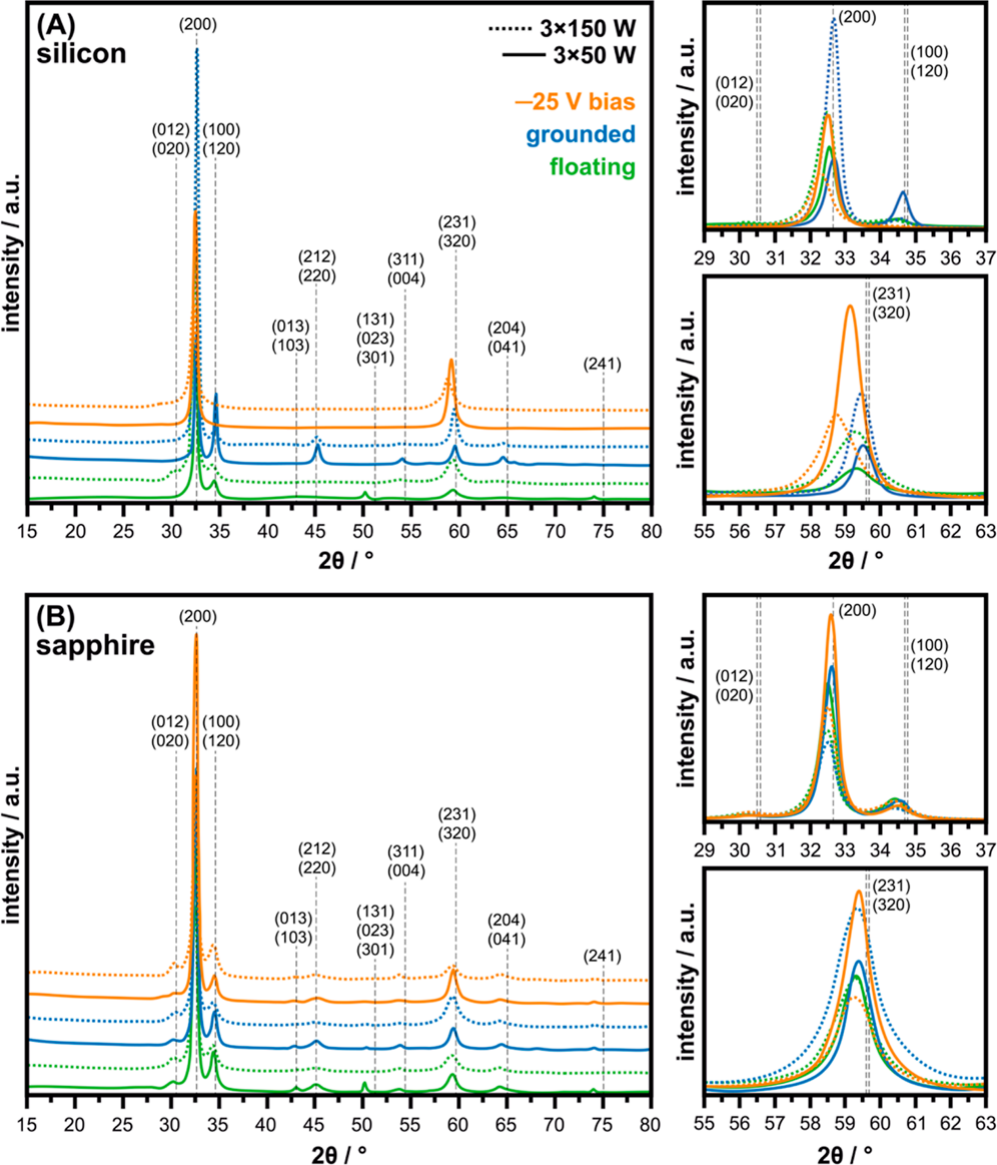
X-ray diffractograms of ZTN films *via* MAR-HiPIMS under varying MW power and substrate bias, dashed grey lines mark the ZnSnN_2_ (orthorhombic/Pna2_1_) non-stressed positions,^40^ (A) silicon and (B) sapphire substrates.

A total of 12 ZTN films were fabricated *via* MAR-HiPIMS. All films were characterised by a chemical composition of approximately Zn_0.92_Sn_1.08_N_2_ (*via* EDX). XRD was performed to further verify the formation of ZTN and determine the deposited materials’ structure (Figure 5). All obtained peaks are related to those of stoichiometric (ZnSnN_2_, orthorhombic/Pna2_1_).^40^ The structures of the films deposited onto Si substrates show clear changes with changing substrate bias and MW power, despite their similar chemical composition. Exact peak identification is hindered due to the complexity of ZTN’s structure, as often two orientations are assigned to a single peak due to possible peak overlapping. However, the general trend is that the material appears more textured with the substrate bias progression and higher MW powers (floating E/Q_ion_ = 5 and 7 V) → grounded (E/Q_ion_ = 10 and 12 V) → −25 V (E/Q_ion_ = 35 and 37 V). For ZTN deposited with a bias of −25 V, already 3 × 50 W were enough to reduce the number of attained orientations to two, with the dominant orientation being (002) at approximately 32.50° and the other (231/320) at 59.16°. Most probably, this sample is characterised by a bi-fiber texture.^41^ The film produced at higher MW powers of 3 × 150 W was very similar to its 3 × 50 W counterpart, however, it is possible to notice a faint shoulder of orientation (012/020) appearing at 30.18°. This could indicate that, under higher MW plasma conditions, random orientations are introduced into the material. When analysing the films deposited onto sapphire substrates, the same trends are not seen. Although the sample holder was subjected to biasing, sapphire is non-conductive, therefore, the incoming ions would not have the same energies as in the case of conductive Si substrates. The diffractograms resemble one another, however, some changes can be distinguished for the ZTN films produced at higher MW powers (higher plasma potentials), *e.g.*, varying peak intensities and slight shifting of the peaks indicating different internal compressive stresses.

**Figure 6:**
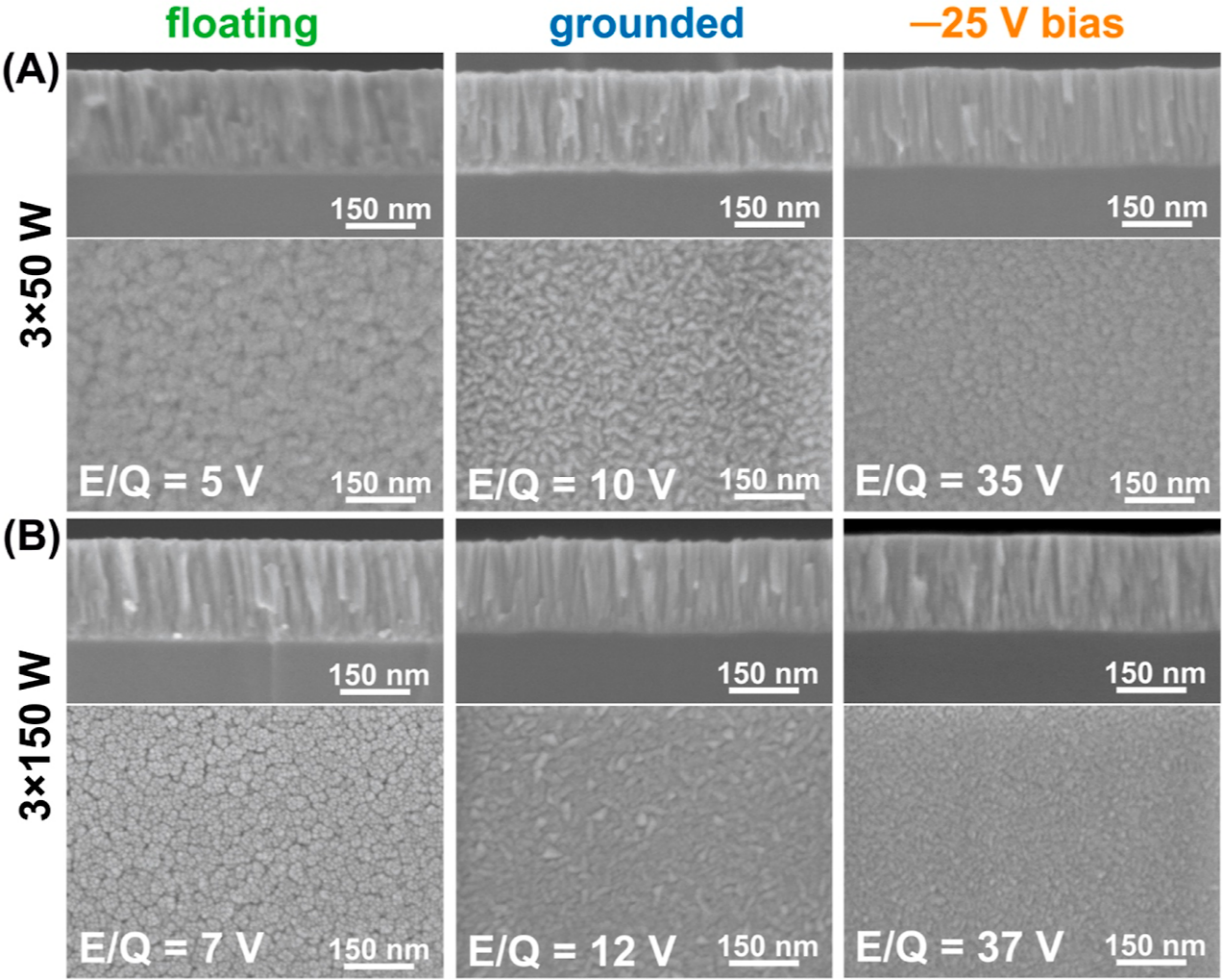
SEM images of fracture cross-sections and surface morphologies of ZTN films deposited onto floating, grounded, and −25 V biased Si substrates under (A) 3 × 50 W and (B) 3 × 150 W MW powers.

Next, the surface and the fracture surface of the ZTN films deposited onto Si were imaged (Figure 6) to determine their thickness, as well as their microstructure and morphology. All samples possessed a homogeneous thickness of approximately 200 nm and exhibited a columnar-type growth. The films deposited at floating and grounded potentials possessed discontinuous and tapered columns (for more details, see Supporting Information, Figure S5). This is additionally reflected by the films’ surface morphology (cauliflower/mudcrack-like for floating potential and rice grain-like for grounded) containing pores. The SEM images also show that higher applied microwave powers (and higher plasma potentials) lead to straighter columns, with finer surface features. Films fabricated at −25 V possess continuous columns through the film, and their surfaces are devoid of noticeable porosities. The difference between the films fabricated using the two applied MW powers is minimal (slightly smaller surface morphology features for the 3 × 150 W variant), as here the increase in plasma potential is slight compared to the applied substrate bias.

**Figure 7:**
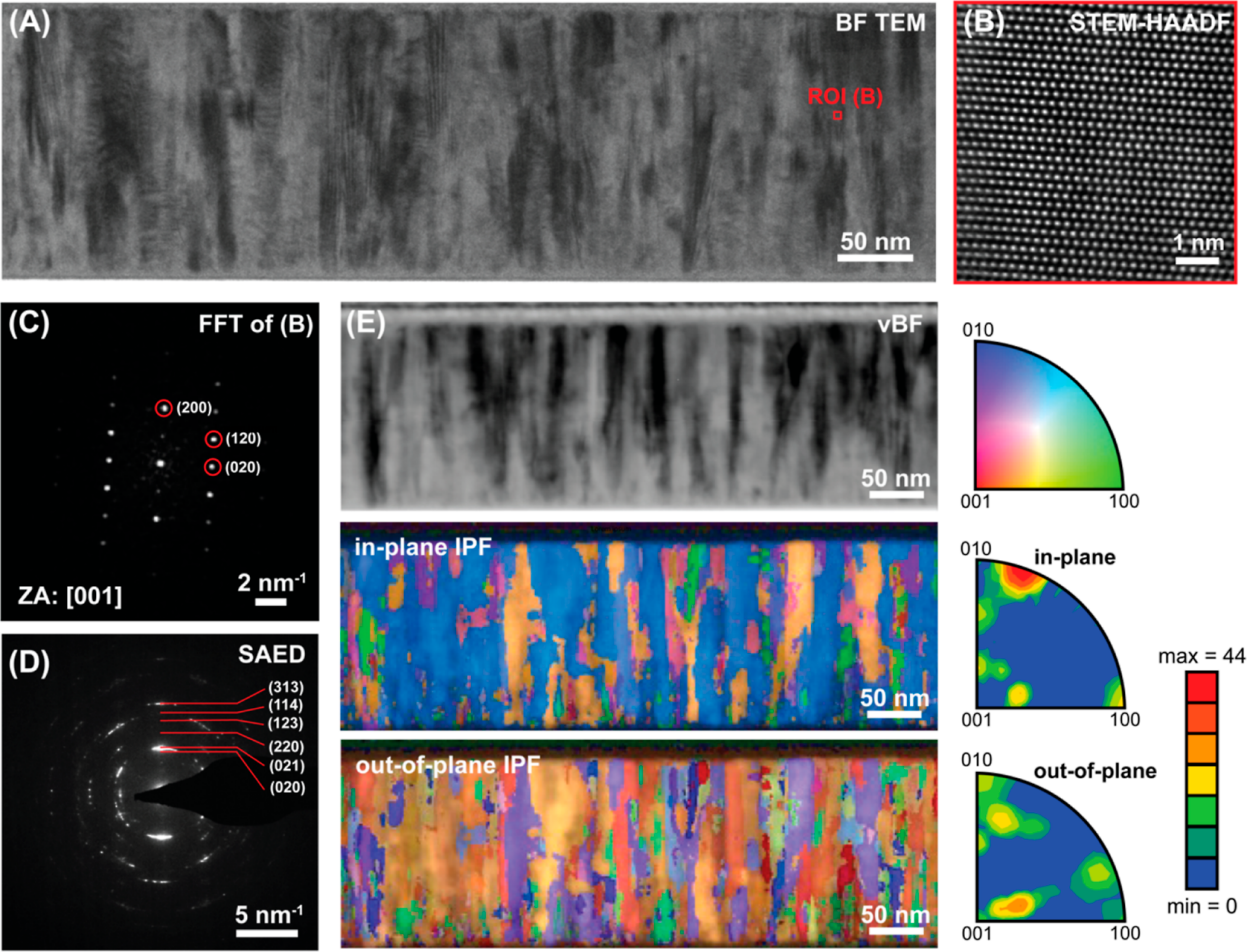
TEM analysis of the film deposited on Si using −25 V bias and 3 × 50 W MW plasma power, (A) BF TEM image showing the overall film structure, (B) chosen ROI (highlighted in red) examined *via* HAADF-STEM, and (C) fast Fourier transformation, (D) SAED pattern collected over a circular area of approximately 150 nm diameter, and (E) SPED analysis performed with a 3 nm step size, where the data collected is showcased through a vBF image and its respective IPF maps.

The film deposited at −25 V bias and 3 × 50 W MW plasma power, showing the most promise in terms of preferential growth and morphology homogeneity, was subjected to more in-depth analysis *via* TEM (Figure 7). From the brightfield (BF) TEM image (Figure 7A), the ZTN film’s columnar structure is confirmed. From there, a region of interest (ROI) was selected, and HAADF-STEM was performed (Figure 7B,C). Furthermore, selected area electron diffraction (SAED, Figure 7D) and SPED (Figure 7E) analyses were performed to characterise the film’s texturing. The SAED pattern contains information averaged over a circular area approximately 150 nm in diameter, while SPED allows to establish the orientation of individual grains, with the virtual brightfield (vBF) image showing the analysed area and the corresponding inverse pole figure (IPF) maps. All detected orientations were identified as belonging to the ZnSnN_2_ phase, and the film showed a high degree of texture in the in-plane (010) direction, in agreement with the XRD results.

**Figure 8:**
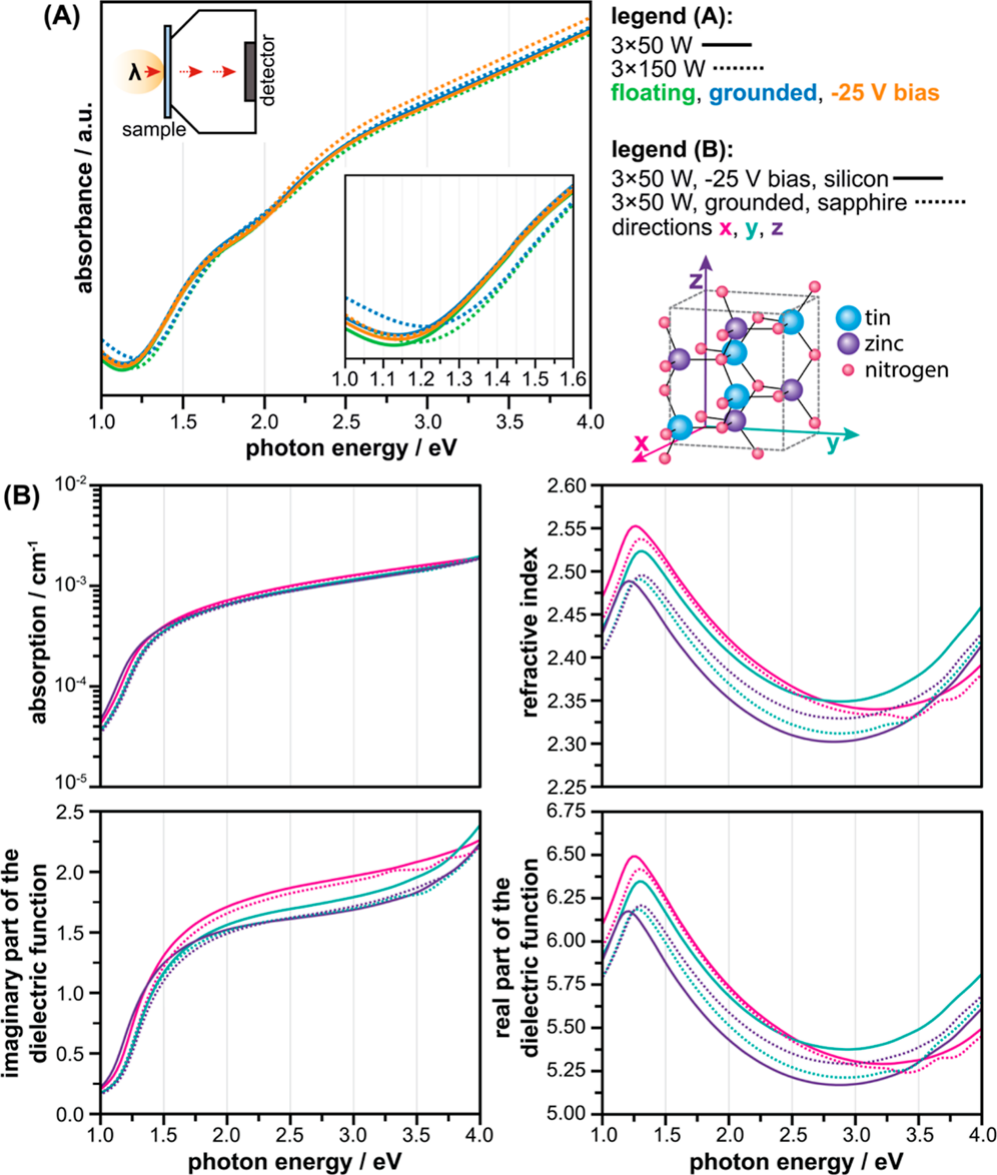
Optical properties of ZTN films (A) experimental, deposited onto sapphire substrates showing the materials’ absorbance as a function of photon energy, including inserts of measurement configuration (top left) and magnified absorption band (bottom right), (B) model DFT calculations using ZTN unit cells with lattice parameters (Supporting Information, Table S2) determined from X-ray diffractograms from two samples deposited onto silicon (3 × 50 W, −25 V) and sapphire (3 × 50 W, 0 V) substrates.

To evaluate the optical properties of the ZTN films, absorption spectrometry measurements were performed (Figure 8A). It was determined that all ZTN films deposited onto sapphire substrates were characterised by an absorption drop at approximately 1.2 eV, as expected for mid-gap semiconductor similar materials (II-IV-N_2_).^22,42^ Upon closer inspection of the films’ absorption band energy, absorption onset ranged between 1.127 and 1.219 eV. To further elucidate the influence of the ZTN films’ structure on their optical properties, computational investigations were performed within the DFT framework. The aim of these calculations was to assess the impact of crystal lattice parameters and the texture of the fabricated ZTN films on their optical properties. The structures used in the DFT calculations were constructed using the lattice parameters derived from two ZTN samples with distinctly different X-ray diffractograms (Figure 5), *i.e.*, 3 × 50 W, −25 V, deposited onto silicon and 3 × 50 W, grounded, deposited onto sapphire. The results for the films’ absorption (α), refractive index (*n*), and imaginary (ε_i_) and real (ε_r_) parts of the dielectric function in the *x*, *y*, and *z* main crystal directions are presented in (Figure 8B). Both structures exhibit optical anisotropy in the *x*, *y*, and *z* directions. However, the calculated values for the two samples varied only slightly.

## 4 Discussion

In terms of sputtering behaviour, Zn and Sn behave similarly to In under R-HiPIMS conditions, where target poisoning can cause a decrease in γ_see_, contributing to the formation of time lags.^14,16^ In MAR-HiPIMS, the MW plasma acts as a source of seed electrons for the magnetron discharge, allowing the negative effects of compound formation on the target surface to be decoupled. Varying the MW plasma power also gives the freedom to modify the properties (*V*_p_, *kT*_e_, *n*_i_, *n*_e_) of the “volume” plasma. This includes not only the ability to influence nitrogen activation (ionisation/excitation) but also to reduce the energy range of incoming ionic species compared to pure HiPIMS. This provides the opportunity to control the behaviour of the charged species, *e.g.*, by altering the overall plasma potential by changing the applied power and/or substrate biasing strategy.

The benefits of these phenomena are evident in several areas. Firstly, it facilitates the formation of ionic metallic nitride detectable in the gas phase, *i.e.*, SnN^+^, SnN_2_^+^, ZnN^+^, and ZnN_2_^+^. The formation of metallic nitrides during MAR-HiPIMS has also been reported in ref (43), where ScN^+^ ions were detected during the fabrication of AlScN films. However, without the presence of MW plasma, *i.e.*, during a pure R-HiPIMS process, Zn and Sn ionic nitrides were not detected. This suggests that not enough reactive nitrogen was generated within the localised HiPIMS plasma and/or that the MW plasma caused the post-ionisation of neutral molecules. One possibility for attaining more nitrogen species would be to increase the nitrogen flow. However, this would further increase target poisoning, which would cause significant sputtering instabilities, as discussed in more detail in our previous work.^15^ This brings us to the second point of creating stoichiometric ZTN films for all deposition series by generating substantial amounts of reactive nitrogen in the MW plasma. The ECR effect facilitates this process, in which electrons are trapped by the electromagnetic field and accelerated to attain energies high enough to excite, ionise, and dissociate nitrogen.^11,44^ Moreover, Figures 3 and 4 (and Figures S1 and S2) demonstrate that with the use of MW plasma in volume, most of the charged species acquire comparable energies, in turn providing the means to control their overall behaviour for obtaining various film textures. Conversely, in the absence of MW plasma, the ions’ energies scatter more extensively (Figures S3 and S4). Ions with energies at either end of the range induce diverse growth effects upon reaching the substrate. Anders’s extended version of the structural zone diagram, encompassing plasma-based phenomena, explains these effects in detail.^45^

The X-ray diffractograms (Figure 5) reflect the impact of various ion energies. For sapphire substrates, which are non-conductive, altering the bias voltage did not have a significant effect on the texture of the films (Figure 5B). When depositing films onto silicon substrates, applying substrate biasing did influence the energies of the incident ions considerably, resulting in a transition from a polycrystalline film for the unbiased (floating) variant to a more preferentially bi-fiber textured film for the −25 V biased substrate (Figures 5A and 7). This can be explained by the substrate biasing increasing adatom mobility on the substrate’s surface.^45−47^ In comparison to the alteration in bias voltage that caused E/Q changes in steps of 5 and 25 V during substrate grounding and biasing, respectively, the change in microwave plasma power from 3 × 50 W to 3 × 150 W caused a difference in E/Q of only 2 V. Therefore, it is not surprising that this induced only small changes in the texture of the ZTN thin films. However, upon examination of the films deposited onto −25 V-biased silicon, it appears that the increase in E/Q from 35 V for 3 × 50 W to 37 V for 3 × 150 W surpassed a certain threshold, possibly starting to introduce random orientations in the material. This is demonstrated by the occurrence of an additional peak in the diffractogram, the formation of smaller grains evidenced by the diffractograms as peak broadening, as well as surface morphology studies (Figure 6). Both SEM and TEM cross-sectional analyses of the films showed that all ZTN films adopted a columnar grain morphology (Figures 6, 7, and S5 of Supporting Information). The ZTN film with the most parallel columnar structure with respect to the substrate surface was found to possess a high level of in-plane texture in the (010) direction (Figure 7). These findings demonstrate that the investigated approach is promising for the determination of the E/Q window for structure tailoring and for the prevention of sample degradation. This method could also be potentially extended to targeted interface engineering of multilayer samples.

Through a combination of first principle calculations and experimental investigations, it was possible to determine the optical anisotropic nature of ZTN. Despite differences in crystallinity resulting from varying deposition parameters and substrate materials, both experimental and computational analyses showed that the optical properties remained relatively constant (Figure 8). The slight modifications are attributed to small variances in the lattice parameters (*a*, *b*, and *c*) of the ZTN films. These may stem from varying degrees of internal compressive stress,^48^ however, they were not quantified within the scope of this paper. The difference between the two chosen film samples, consisting of 3 × 50 W, −25 V, deposited onto silicon and 3 × 50 W, grounded, deposited onto sapphire, was found to be less than 0.02 Å, according to Rietveld refinement of the films’ X-ray diffractograms (see Supporting Information, Table S2). Shohonov *et al.* have demonstrated how even minor variations in lattice parameters can impact a material’s properties, as evidenced by their study of BaSi_2_ films,^49^ where a change in *a* as small as 0.03 Å resulted in a change in bandgap of approximately 0.08 eV. In our study, the experimentally measured change in absorption onset edge ranged to approximately 0.09 eV (Figure 8A).

The impact of structural disorder on the tunability of ZTN films has been a subject of study in literature, with differing outcomes. Quayle *et al.*(50) have reported that disorder has a marginal effect on the bandgap. However, Lany *et al.*(51) demonstrated the possibility of immobilising (freezing) disorder sites in deposited ZTN films, allowing to tune the bandgap as a function of the amount of these “frozen” sites under non-equilibrium synthesis conditions, such as during sputtering. It is possible that continuous exposure to MW plasma and the consequent impact of charged species, possessing a narrow energy range in contrast to classical sputtering, alter the range of cooling rates. This, in turn, may prevent the rapid cooling and confinement of disordered sites within the ZTN structure. However, further investigations into the thermodynamic phenomena at play are necessary to understand this behaviour in detail.

## 5 Conclusions

In this work, ZTN films were produced on two different substrate types using varying microwave plasma power and substrate bias conditions during MAR-HiPIMS deposition. The obtained results indicate that:

- Regardless of the parameters and substrates utilised, stoichiometric ZTN films were achieved with similar chemical compositions, confirming the suitability of MAR-HiPIMS for nitride production.
- Microwave plasma assists in creating ionic nitrides in the gas phase, post-ionising sputtered atomic species, and narrowing the E/Q range of incoming ions, thus enabling improved control over texture tailoring.
- The sputtered ZTN films exhibited optical anisotropy, however, their optical properties are comparable overall due to only minor changes in the lattice parameters of the materials, despite significant differences in their crystallinity.

Further research is needed to characterise the thermodynamic processes involved in MAR-HiPIMS. Nevertheless, the potential of MAR-HiPIMS for other optics-related materials, beyond ZTN, is promising, as demonstrated by the work of Mitterer *et al.* on titanium and zirconium nitrides,^52^ which highlighted the importance of the uniformity of ion energy distribution in controlling the stoichiometry and lattice parameters of these materials and consequently their optical properties (or indeed other structure-dependent properties). Additionally, the possibility of tailoring film texturing through *in situ* deposition environment analysis holds great promise for the production of multilayer devices, *e.g.*, in the context of photovoltaics, where precise control over the structure and microstructure, as well as interface engineering, is crucial for enhancing overall device performance.

## Supporting Information Available

Additional experimental results and calculations, including Langmuir probe analysis results, E-ToFMS contour plots, TEM images, and lattice parameter constants (PDF)

## References

[ref1] Moatti A.; Sachan R.; Prater J.; Narayan J. Control of Structural and Electrical Transitions of VO2 Thin Films. ACS Appl. Mater. Interfaces 2017, 9 (28), 24298–24307. 10.1021/acsami.7b05620. 28622721

[ref2] Patidar J.; Sharma A.; Zhuk S.; Lorenzin G.; Cancellieri C.; Sarott M. F.; Trassin M.; Thorwarth K.; Michler J.; Siol S. Improving the Crystallinity and Texture of Oblique-Angle-Deposited AlN Thin Films Using Reactive Synchronized HiPIMS. Surf. Coat. Technol. 2023, 468, 129719 10.1016/j.surfcoat.2023.129719.

[ref3] Mickan M.; Helmersson U.; Rinnert H.; Ghanbaja J.; Muller D.; Horwat D. Room Temperature Deposition of Homogeneous, Highly Transparent and Conductive Al-Doped ZnO Films by Reactive High Power Impulse Magnetron Sputtering. Sol. Energy Mater. Sol. Cells 2016, 157, 742 10.1016/j.solmat.2016.07.020.

[ref4] Mascaretti L.; Barman T.; Bricchi B. R.; Münz F.; Li Bassi A.; Kment S.; Naldoni A. Controlling the Plasmonic Properties of Titanium Nitride Thin Films by Radiofrequency Substrate Biasing in Magnetron Sputtering. Appl. Surf. Sci. 2021, 554, 149543 10.1016/j.apsusc.2021.149543.

[ref5] Lee H. Y.; Al Ezzi M. M.; Raghuvanshi N.; Chung J. Y.; Watanabe K.; Taniguchi T.; Garaj S.; Adam S.; Gradečak S. Tunable Optical Properties of Thin Films Controlled by the Interface Twist Angle. Nano Lett. 2021, 21 (7), 2832–2839. 10.1021/acs.nanolett.0c04924. 33591206

[ref6] Kouznetsov V.; Macák K.; Schneider J. M.; Helmersson U.; Petrov I. A Novel Pulsed Magnetron Sputter Technique Utilizing Very High Target Power Densities. Surf. Coat. Technol. 1999, 122 (2–3), 290–293. 10.1016/S0257-8972(99)00292-3.

[ref7] Anders A. Tutorial: Reactive High Power Impulse Magnetron Sputtering (R-HiPIMS). J. Appl. Phys. 2017, 121 (17), 1–34. 10.1063/1.4978350.

[ref8] Lundin D.; Gudmundsson J. T.; Minea T. High Power Impulse Magnetron Sputtering: Fundamentals, Technologies, Challenges and Applications; Elsevier, 2019.

[ref9] Gudmundsson J. T.; Brenning N.; Lundin D.; Helmersson U. High Power Impulse Magnetron Sputtering Discharge. J. Vac. Sci. Technol., A 2012, 30 (3), 030801 10.1116/1.3691832.

[ref10] Latrasse L.; Radoiu M.; Nelis T.; Antonin O. Self-Matching Plasma Sources Using 2.45 GHz Solid-State Generators: Microwave Design and Operating Performance. J. Microw. Power Electromagn. Energy 2017, 51 (4), 237–258. 10.1080/08327823.2017.1388338.

[ref11] Zoubian F.; Renaut N.; Latrasse L. Distributed Elementary ECR Microwave Plasma Sources Supplied by Solid State Generators for Production of Large Area Plasmas without Scale Limitation: Plasma Density Measurements and Comparison with Simulation. Plasma Res. Express 2021, 3 (2), 025010 10.1088/2516-1067/ac0499.

[ref12] Depla D.; Mahieu S. Reactive Sputter Deposition; Depla D., Mahieu S., Eds.; Springer Series in Materials Science; Springer Berlin Heidelberg: Berlin, Heidelberg, 2008; Vol. 109.

[ref13] Güttler D.; Abendroth B.; Grötzschel R.; Möller W.; Depla D. Mechanisms of Target Poisoning during Magnetron Sputtering as Investigated by Real-Time in Situ Analysis and Collisional Computer Simulation. Appl. Phys. Lett. 2004, 85 (25), 6134–6136. 10.1063/1.1835002.

[ref14] Depla D.; Mahieu S.; De Gryse R. Magnetron Sputter Deposition: Linking Discharge Voltage with Target Properties. Thin Solid Films 2009, 517 (9), 2825–2839. 10.1016/j.tsf.2008.11.108.

[ref15] Hain C.; Schweizer P.; Sturm P.; Borzì A.; Thomet J. E.; Michler J.; Hessler-Wyser A.; Nelis T. Microwave Plasma-Assisted Reactive HiPIMS of InN Films: Plasma Environment and Material Characterisation. Surf. Coat. Technol. 2023, 454, 129188 10.1016/j.surfcoat.2022.129188.

[ref16] Yushkov G. Y.; Anders A. Origin of the Delayed Current Onset in High-Power Impulse Magnetron Sputtering. IEEE Trans. Plasma Sci. 2010, 38, 3028–3034. 10.1109/TPS.2010.2063041.

[ref17] Lahourcade L.; Coronel N. C.; Delaney K. T.; Shukla S. K.; Spaldin N. A.; Atwater H. A. Structural and Optoelectronic Characterization of RF Sputtered ZnSnN2. Adv. Mater. 2013, 25 (18), 2562–2566. 10.1002/adma.201204718. 23386387

[ref18] Alnjiman F.; Diliberto S.; Ghanbaja J.; Haye E.; Kassavetis S.; Patsalas P.; Gendarme C.; Bruyère S.; Cleymand F.; Miska P.; Boulet P.; Pierson J. F. Chemical Environment and Functional Properties of Highly Crystalline ZnSnN2 Thin Films Deposited by Reactive Sputtering at Room Temperature. Sol. Energy Mater. Sol. Cells 2018, 182, 30–36. 10.1016/j.solmat.2018.02.037.

[ref19] Chen S.; Narang P.; Atwater H. A.; Wang L.-W.; Chen S.; Narang P.; Atwater H. A.; Wang L.-W.; Watson T. J. Phase Stability and Defect Physics of a Ternary ZnSnN2 Semiconductor: First Principles Insights. Adv. Mater. 2014, 26 (2), 311–315. 10.1002/ADMA.201302727. 24403116

[ref20] Paudel T. R.; Lambrecht W. R. L. First-Principles Study of Phonons and Related Ground-State Properties and Spectra in Zn-IV-N2 Compounds. Phys. Rev. B: Condens. Matter Mater. Phys. 2008, 78 (11), 115204 10.1103/physrevb.78.115204.

[ref21] Chinnakutti K. K.; Panneerselvam V.; Thankaraj Salammal S. Tailoring Optoelectronic Properties of Earth Abundant ZnSnN2 by Combinatorial RF Magnetron Sputtering. J. Alloys Compd. 2019, 772, 348–358. 10.1016/j.jallcom.2018.08.331.

[ref22] Fioretti A. N.; Zakutayev A.; Moutinho H.; Melamed C.; Perkins J. D.; Norman A. G.; Al-Jassim M.; Toberer E. S.; Tamboli A. C. Combinatorial Insights into Doping Control and Transport Properties of Zinc Tin Nitride. J. Mater. Chem. C Mater. 2015, 3 (42), 11017–11028. 10.1039/C5TC02663F.

[ref23] Djamila H.; Belkhir H.; Ali S.; Bououdina M. In-Depth Analysis of Ternary NaCuX (X = Se and Te) Chalcogenides: Bridging Structural Elastic, Electronic, and Optical Properties. Mater. Today Commun. 2023, 37, 107426 10.1016/j.mtcomm.2023.107426.

[ref24] Gu Y.; Cai H.; Dong J.; Yu Y.; Hoffman A. N.; Liu C.; Oyedele A. D.; Lin Y. C.; Ge Z.; Puretzky A. A.; Duscher G.; Chisholm M. F.; Rack P. D.; Rouleau C. M.; Gai Z.; Meng X.; Ding F.; Geohegan D. B.; Xiao K. Two-Dimensional Palladium Diselenide with Strong In-Plane Optical Anisotropy and High Mobility Grown by Chemical Vapor Deposition. Adv. Mater. 2020, 32 (19), 1–10. 10.1002/adma.201906238. 32173918

[ref25] Moussaoui I.; Kadri M. T.; Belkhir H.; Bououdina M. Comparative First-Principles Calculations of Structural, Elastic, Electronic and Optical Properties of Orthorhombic Inter-Alkali Metal Chalcogenides NaLiSe and NaLiTe. Eur. Phys. J. Plus 2022, 137, 1055 10.1140/epjp/s13360-022-03271-9.

[ref26] Ransmayr V.; Tomczak J. M.; Galler A. Relation between Crystal Structure and Optical Properties in the Correlated Blue Pigment YIn_1-X_Mn_x_O_3_. Phys. Rev. Mater. 2022, 6, 105003 10.1103/PhysRevMaterials.6.105003.

[ref27] Latrasse L.; Lacoste A.; Sirou J.; Pelletier J. High Density Distributed Microwave Plasma Sources in a Matrix Configuration: Concept, Design and Performance. Plasma Sources Science and Technology; IOP Publishing, 2007; Vol. 16, pp 7–12.

[ref28] Hain C.; Brown D.; Welsh A.; Wieczerzak K.; Weiss R.; Michler J.; Hessler-Wyser A.; Nelis T. From Pulsed-DCMS and HiPIMS to Microwave Plasma-Assisted Sputtering: Their Influence on the Properties of Diamond-like Carbon Films. Surf. Coat. Technol. 2022, 432, 127928 10.1016/j.surfcoat.2021.127928.

[ref29] Mayer J.; Giannuzzi L. A.; Kamino T.; Michael J. TEM Sample Preparation and FIB-Induced Damage. MRS Bull. 2007, 32, 400–407. 10.1557/mrs2007.63.

[ref30] Madsen G. K. H.; Blaha P.; Schwarz K.; Sjöstedt E.; Nordström L. Efficient Linearization of the Augmented Plane-Wave Method. Phys. Rev. B 2001, 64 (19), 195134 10.1103/PhysRevB.64.195134.

[ref31] Kohn W.; Sham L. J. Self-Consistent Equations Including Exchange and Correlation Effects. Phys. Rev. 1965, 140 (4A), A1133–A1138. 10.1103/physrev.140.a1133.

[ref32] Hohenberg P.; Kohn W. Inhomogeneous Electron Gas. Phys. Rev. 1964, 136 (3B), B864–B871. 10.1103/physrev.136.b864.

[ref33] Blaha P.; Schwarz K.; Tran F.; Laskowski R.; Madsen G. K. H.; Marks L. D. WIEN2k: An APW+lo Program for Calculating the Properties of Solids. J. Chem. Phys. 2020, 152 (7), 74101 10.1063/1.5143061. 32087668

[ref34] Perdew J. P.; Burke K.; Ernzerhof M. Generalized Gradient Approximation Made Simple. Phys. Rev. Lett. 1996, 77 (18), 3865–3868. 10.1103/PhysRevLett.77.3865. 10062328

[ref35] Tran F.; Blaha P. Accurate Band Gaps of Semiconductors and Insulators with a Semilocal Exchange-Correlation Potential. Phys. Rev. Lett. 2009, 102 (22), 226401 10.1103/physrevlett.102.226401. 19658882

[ref36] Tran F.; Doumont J.; Kalantari L.; Huran A. W.; Marques M. A. L.; Blaha P. Semilocal Exchange-Correlation Potentials for Solid-State Calculations: Current Status and Future Directions. J. Appl. Phys. 2019, 126 (11), 110902 10.1063/1.5118863.

[ref37] Ambrosch-Draxl C.; Sofo J. O. Linear Optical Properties of Solids within the Full-Potential Linearized Augmented Planewave Method. Comput. Phys. Commun. 2006, 175 (1), 1–14. 10.1016/j.cpc.2006.03.005.

[ref38] Coelho A. A. TOPAS: General Profile and Structure Analysis Software for Powder Diffraction Data; Bruker AXS GmbH: Karlsruhe, Germany, 2003.

[ref39] Rietveld H. M. A Profile Refinement Method for Nuclear and Magnetic Structures. J. Appl. Crystallogr. 1969, 2 (2), 65–71. 10.1107/S0021889869006558.

[ref40] Jain A.; Ong S. P.; Hautier G.; Chen W.; Richards W. D.; Dacek S.; Cholia S.; Gunter D.; Skinner D.; Ceder G.; Persson K. A. Commentary: The Materials Project: A Materials Genome Approach to Accelerating Materials Innovation. APL Mater. 2013, 1 (1), 011002 10.1063/1.4812323.

[ref41] Sharma A.; Mohan S.; Suwas S. The Effect of the Deposition Rate on the Crystallographic Texture, Microstructure Evolution and Magnetic Properties in Sputter Deposited Ni-Mn-Ga Thin Films. Thin Solid Films 2016, 616, 530–542. 10.1016/j.tsf.2016.08.033.

[ref42] Greenaway A. L.; Ke S.; Culman T.; Talley K. R.; Mangum J. S.; Heinselman K. N.; Kingsbury R. S.; Smaha R. W.; Gish M. K.; Miller E. M.; Persson K. A.; Gregoire J. M.; Bauers S. R.; Neaton J. B.; Tamboli A. C.; Zakutayev A. Zinc Titanium Nitride Semiconductor toward Durable Photoelectrochemical Applications. J. Am. Chem. Soc. 2022, 144 (30), 13673–13687. 10.1021/jacs.2c04241. 35857885 PMC9354241

[ref43] Lapeyre L.; Hain C.; Sturm P.; Metzger J.; Borzì A.; Wieczerzak K.; Raynaud P.; Michler J.; Nelis T. Deposition and Characterisation of C-Axis Oriented AlScN Thin Films via Microwave Plasma-Assisted Reactive HiPIMS. Surf. Coat. Technol. 2023, 464, 129540 10.1016/j.surfcoat.2023.129540.

[ref44] Ruchkina M.; Hot D.; Ding P.; Hosseinnia A.; Bengtsson P. E.; Li Z.; Bood J.; Sahlberg A. L. Laser-Induced Thermal Grating Spectroscopy Based on Femtosecond Laser Multi-Photon Absorption. Sci. Rep. 2021, 11 (1), 9829 10.1038/s41598-021-89269-2. 33972614 PMC8110561

[ref45] Anders A. A Structure Zone Diagram Including Plasma-Based Deposition and Ion Etching. Thin Solid Films 2010, 518 (15), 4087–4090. 10.1016/j.tsf.2009.10.145.

[ref46] Chia R. W. J.; Wang C. C.; Lee J. J. K. Effect of Adatom Mobility and Substrate Finish on Film Morphology and Porosity: Thin Chromium Film on Hard Disk. J. Magn. Magn. Mater. 2000, 209 (1–3), 45–49. 10.1016/S0304-8853(99)00642-3.

[ref47] Lee H. C.; Lee J. Y.; Ahn H. J. Effect of the Substrate Bias Voltage on the Crystallographic Orientation of Reactively Sputtered AlN Thin Films. Thin Solid Films 1994, 251, 136–140. 10.1016/0040-6090(94)90678-5.

[ref48] Kumar R.; Khare N.; Kumar V.; Bhalla G. L. Effect of Intrinsic Stress on the Optical Properties of Nanostructured ZnO Thin Films Grown by Rf Magnetron Sputtering. Appl. Surf. Sci. 2008, 254 (20), 6509–6513. 10.1016/j.apsusc.2008.04.012.

[ref49] Shohonov D. A.; Migas D. B.; Filonov A. B.; Borisenko V. E.; Takabe R.; Suemasu T. Effects of Lattice Parameter Manipulations on Electronic and Optical Properties of BaSi2. Thin Solid Films 2019, 686, 137436 10.1016/j.tsf.2019.137436.

[ref50] Quayle P. C.; Blanton E. W.; Punya A.; Junno G. T.; He K.; Han L.; Zhao H.; Shan J.; Lambrecht W. R. L.; Kash K. Charge-Neutral Disorder and Polytypes in Heterovalent Wurtzite-Based Ternary Semiconductors: The Importance of the Octet Rule. Phys. Rev. B 2015, 91, 205207 10.1103/PhysRevB.91.205207.

[ref51] Lany S.; Fioretti A. N.; Zawadzki P. P.; Schelhas L. T.; Toberer E. S.; Zakutayev A.; Tamboli A. C. Monte Carlo Simulations of Disorder in ZnSnN 2 and the Effects on the Electronic Structure. Phys. Rev. Mater. 2017, 1, 035401 10.1103/PhysRevMaterials.1.035401.

[ref52] Mitterer C.; Mayrhofer P. H.; Waldhauser W.; Kelesoglu E.; Losbichler P. The Influence of the Ion Bombardment on the Optical Properties of TiNx and ZrNx Coatings. Surf. Coat. Technol. 1998, 108–109, 230–235. 10.1016/S0257-8972(98)00651-3.

