# Supplementary material for: Nanostructure and Optical Property Tailoring of Zinc Tin Nitride Thin Films through Phenomenological Decoupling: A Pathway to Enhanced Control
Source: ACS Appl Nano Mater. 2024 Mar 11;7(6):6242–52. doi: 10.1021/acsanm.3c06178 (PMC10964195; doi:10.1021/acsanm.3c06178)
Supplement: Supplementary file 1 — an3c06178_si_001.pdf [file an3c06178_si_001.pdf]

SUPPORTING INFORMATION

Nanostructure and Optical Property Tailoring of Zinc Tin Nitride Thin Films Through Phenomenological Decoupling: A Pathway to Enhanced Control

Caroline Hain [a,b,c,], Krzysztof Wieczerzak [c], Daniele Casari [c], Amit Sharma [c], Angelos Xomalis[c,e], Patrick Sturm [d], Johann Michler [c], Aïcha Hessler-Wyser [b], Thomas Nelis [a,c]*

[a] Institute for Applied Laser, Photonics and Surface Technologies, BFH, Bern University of Applied Sciences, Quellgasse 21, 2502 Biel/Bienne, Switzerland

[b] Laboratory for Photovoltaics and Thin Film Electronics, EPFL, École Polytechnique Fédérale de Lausanne, Rue de la Maladière 71b, 2000 Neuchâtel, Switzerland

[c] Laboratory for Mechanics of Materials and Nanostructures, Empa, Swiss Federal Laboratories for Materials Science and Technology, Feuerwerkerstrasse 39, 3602 Thun, Switzerland

[d] Tofwerk AG, Schorenstrasse 39, 3645 Thun, Switzerland

[e] Nanoelectronics and Photonics Group, Department of Electronic Systems, Norwegian University of Science and Technology, 7034 Trondheim, Norway

* corresponding author

tel.: +41 32 321 67 55

e-mail: caroline.hain@empa.ch

E-ToFMS contour plots under MAR-HiPIMS conditions (3×150 W microwave power)

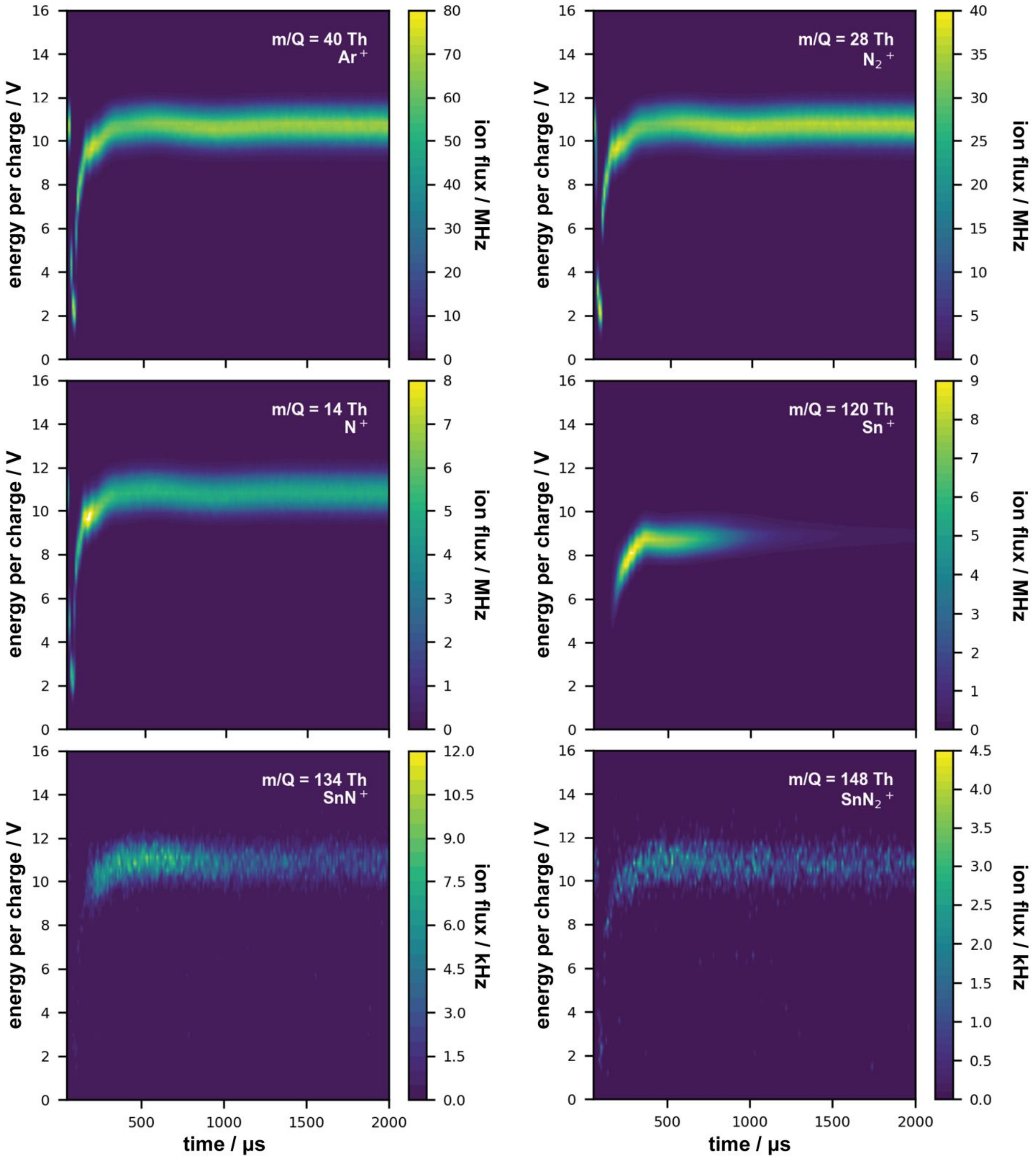

Figure S1 Time-resolved E-ToFMS contour plots for Ar^+, N_2^+, N^+, Sn^+, SnN^+, SnN_2^+ ion fluxes (number of detected ions per unit of time), with their associated E/Q, obtained during MAR-HiPIMS Sn sputtering under Ar/N_2 gas MW plasma conditions (MW power 3×150 W), scales adapted to the data range;

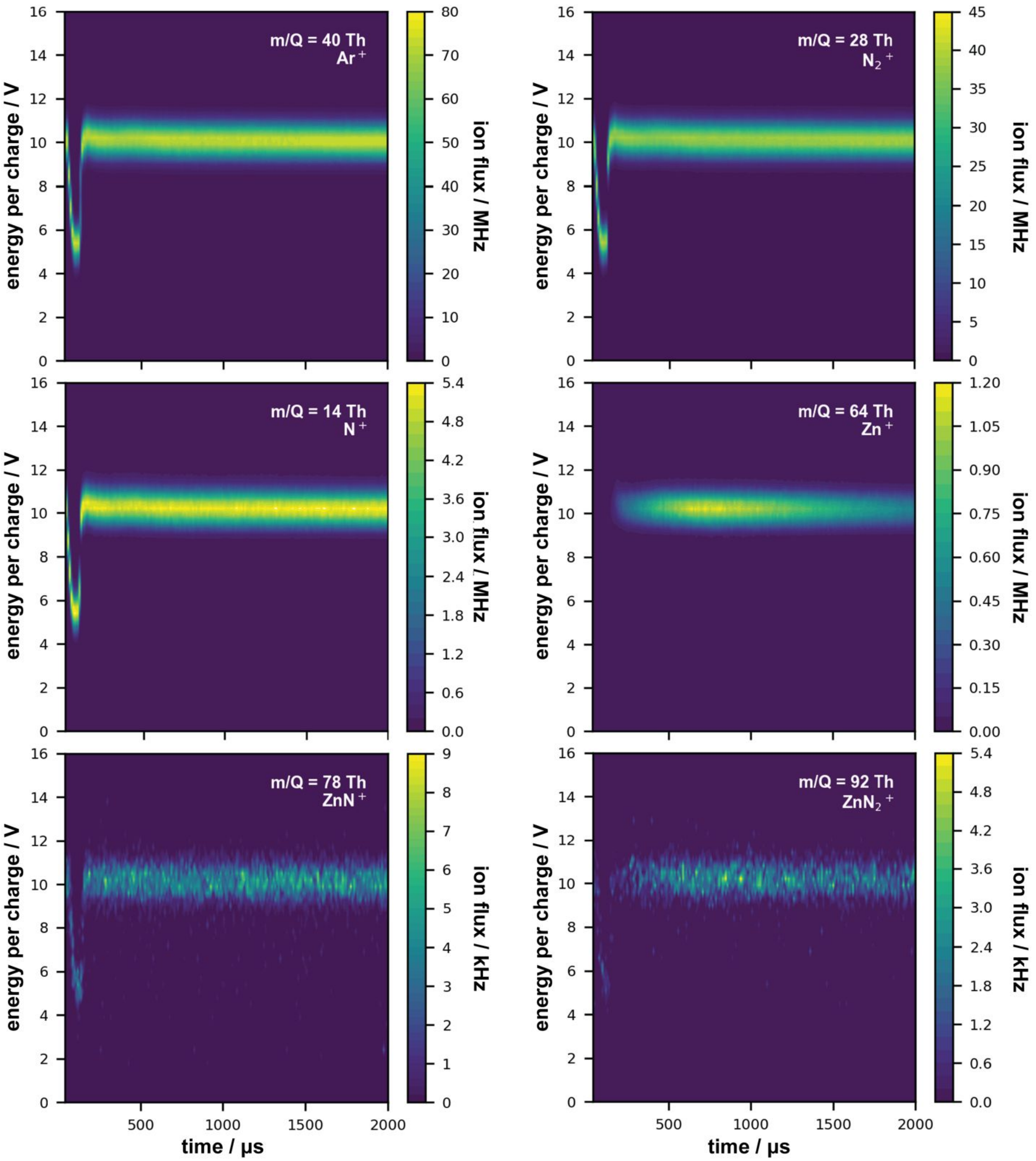

Figure S2 Time-resolved E-ToFMS contour plots for Ar^{+}, N_2^{+}, N^{+}, Zn^{+}, ZnN^{+}, ZnN_2^{+} ion fluxes (number of detected ions per unit of time), with their associated E/Q, obtained during MAR-HiPIMS Zn sputtering under Ar/N_2 gas MW plasma conditions (MW power 3×150 W), scales adapted to the data range;

E-ToFMS contour plots under R-HiPIMS conditions (no MW plasma)

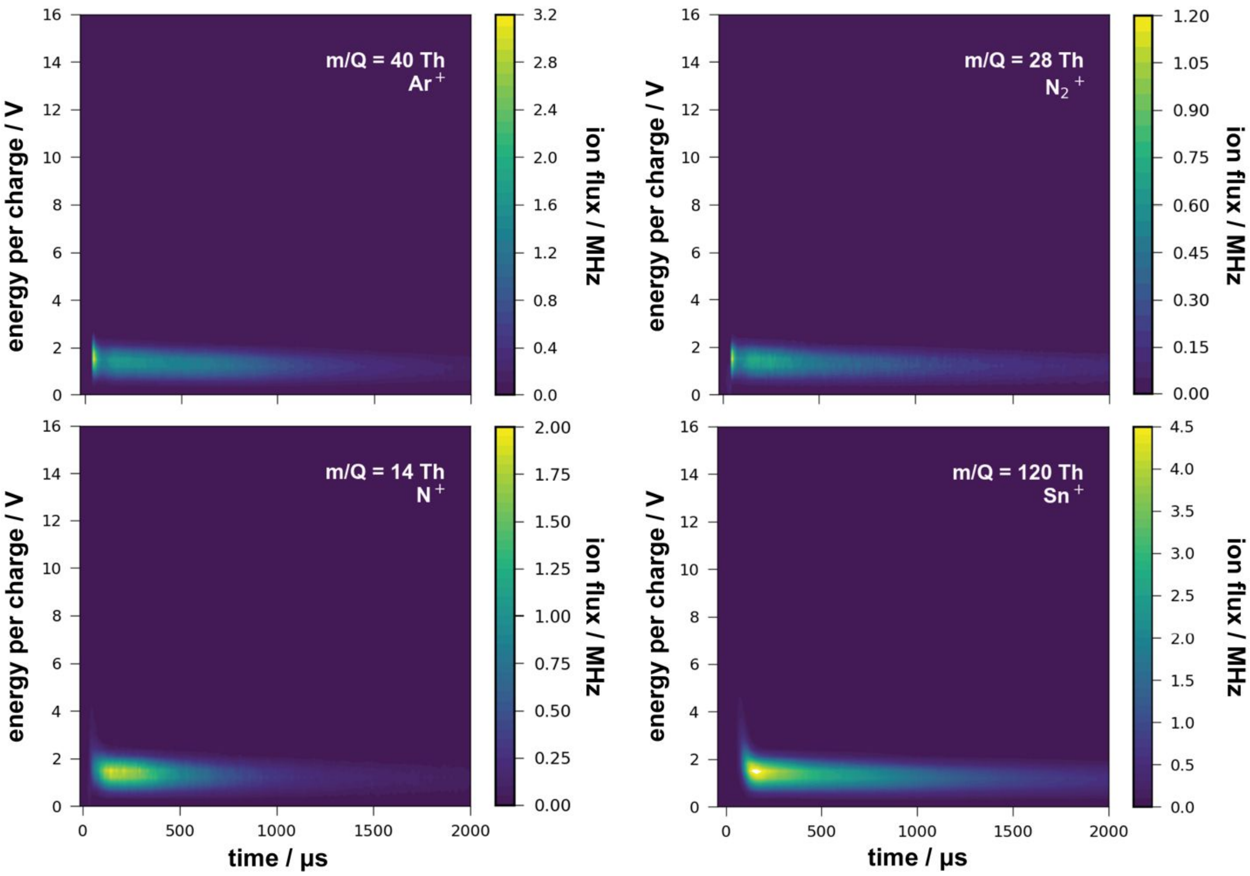

Figure S3 Time-resolved E-ToFMS contour plots for Ar^+, N_2^+, N^+, Sn^+ ion fluxes (number of detected ions per unit of time), with their associated E/Q, obtained during R-HiPIMS Sn sputtering under Ar/N_2 gas conditions, scales adapted to the data range;

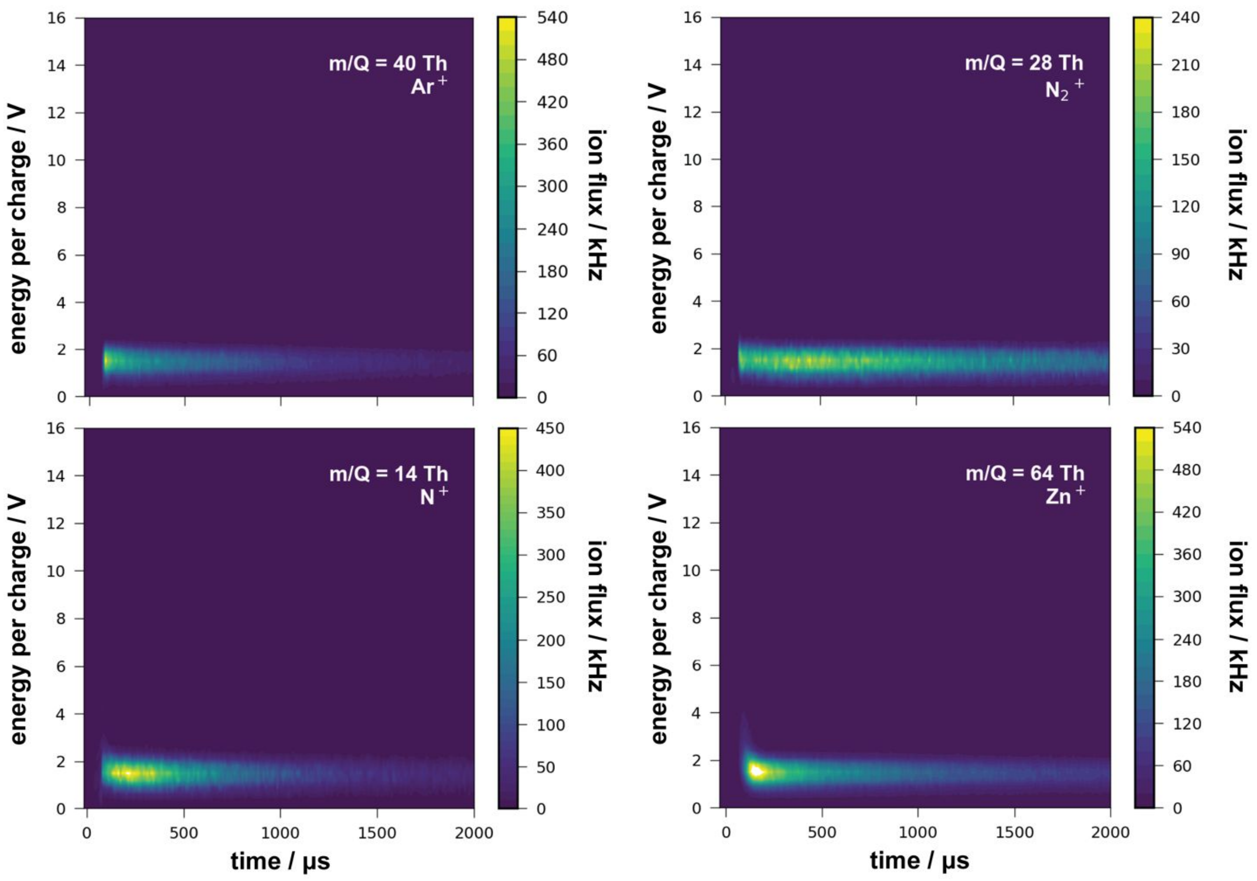

Figure S4 Time-resolved E-ToFMS contour plots for Ar^+, N_2^+, N^+, Zn^+ ion fluxes (number of detected ions per unit of time), with their associated E/Q, obtained during R-HiPIMS Sn sputtering under Ar/N_2 gas conditions, scales adapted to the data range;

Langmuir probe measurements

Table S1 Langmuir probe measurements of V_p, kT_e, n_e, and n_i performed at the substrate region during continuous exposure to an Ar/N_2 (60/40 sccm) MW plasma;

microwave power / W	V_p / V	kT_e / eV	n_e / m^{-3}	n_i / m^{-3}
**3×50**	12	1.4	8×10^{15}	1×10^{16}
**3×150**	14	1.6	1×10^{16}	2×10^{16}

Lattice constants of ZTN films

The Rietveld model was used to determine the lattice constants of two ZTN films, i.e. 3×50 W microwave power and -25 V substrate bias onto a silicon substrate, and 3×50 W microwave power and grounded substrate bias onto a sapphire substrate. Five degrees of freedom were applied to the model, namely 3 lattice parameters (a, b, c; angles were fixed to 90°), crystallite size, and lattice strain. The extracted lattice parameters are given in Table S1.

Table S2 Rietveld-extracted lattice parameters of two ZTN films deposited onto silicon (3×50 W, -25 V) and sapphire (3×50 W, 0 V) substrates;

lattice parameter	3×50 W, -25 V, silicon substrate	3×50 W, 0 V, sapphire substrate
a	5.512800 (Å)	5.496361 (Å)
b	5.894949 (Å)	5.879423 (Å)
c	6.875766 (Å)	6.891292 (Å)

Additional TEM imaging of ZTN

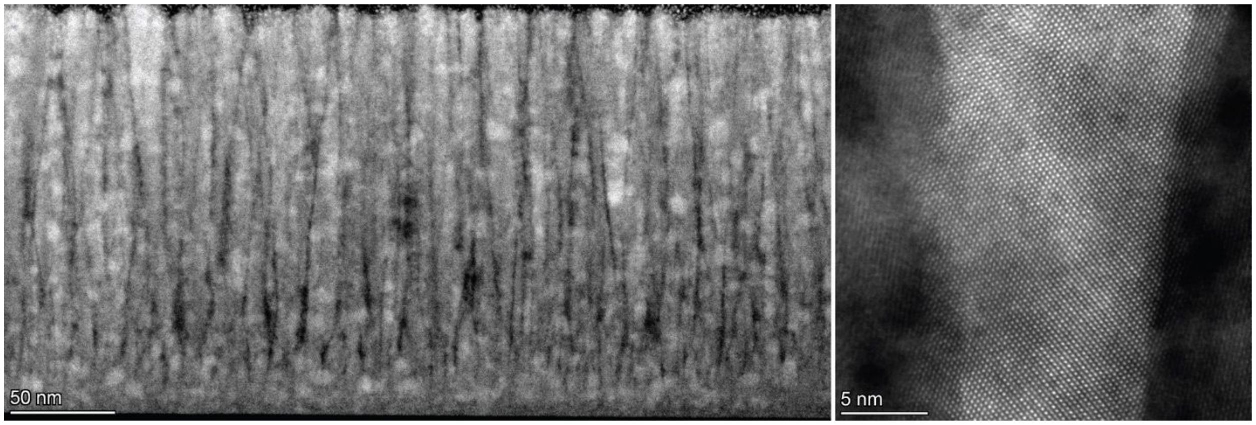

Figure S5 BF TEM image of a ZTN film deposited onto Si at floating potential using 3×50 W MW plasma power;
